# A deep hybrid CSAE-GRU framework with two-stage balancing for automatic epileptic seizure detection using EEG-derived features

**DOI:** 10.3389/fnins.2025.1698960

**Published:** 2025-11-11

**Authors:** Fei Xiang, Mingyue Liu, Wenna Chen, Shaojie Zheng, Jincan Zhang, Ganqin Du

**Affiliations:** 1 College of Information Engineering, Henan University of Science and Technology, Luoyang, China; 2 The First Affiliated Hospital, and College of Clinical Medicine of Henan University of Science and Technology, Luoyang, China

**Keywords:** seizure detection, convolutional neural network, gated recurrent unit, sparse autoencoder, borderline synthetic minority oversampling technique

## Abstract

**Introduction:**

Epilepsy is a neurological disorder characterized by abnormal neuronal discharges in the brain, posing a persistent challenge in clinical diagnosis. This study presents a high-performance epileptic seizure detection framework that integrates advanced feature extraction and classification techniques using EEG signals.

**Methods:**

We conduct experiments on the Bonn and CHB-MIT EEG datasets. The EEG signals are preprocessed through bandpass filtering and five-level Discrete Wavelet Transform (DWT) decomposition. From each sub-band, four representative features are systematically extracted. To mitigate severe class imbalance, we propose a two-stage balancing strategy: cluster centroid-based under-sampling initially reduces the interictal-to-ictal ratio to 2:1, followed by Borderline Synthetic Minority Oversampling Technique (BLSMOTE) in the feature space. A hybrid classification model that combines Convolutional Sparse Autoencoder (CSAE) with Gated Recurrent Unit (GRU) is proposed in the paper. The encoder weights from the pre-trained CSAE are transferred to the GRU-based classifier to enhance feature representation and model generalization.

**Results:**

The proposed method achieves outstanding performance, with accuracy, sensitivity, specificity, precision, f1 score and AUC of 98.46, 98.27, 98.50, 98.36, 98.31, and 98.23% on the Bonn dataset, and 99.49, 99.21, 99.77, 99.49, 99.35, and 99.57% on the CHB-MIT dataset, respectively. These results validate the effectiveness of the proposed approach.

**Discussion:**

This study introduces a novel framework combining cluster centroid-based under-sampling, BLSMOTE oversampling, and transfer learning via CSAE-GRU integration. The method offers a promising direction for reliable and clinically applicable automated epilepsy diagnosis.

## 1 Introduction

Epilepsy, as a prevalent neurological disorder affecting individuals of all ages, arises from paroxysmal abnormal neuronal discharges within the brain. Among neurological disorders, epilepsy ranks second only to cerebrovascular disease in prevalence (Beniczky et al., 2021). Common types of epilepsy encompass focal epilepsy, generalized epilepsy, idiopathic seizure, and secondary seizure (Sethy et al., 2023). Seizure typically entail loss of consciousness accompanied by convulsions, posing risks of irreversible brain damage, shock, and potentially fatal outcomes in severe instances (Sharan and Berkovsky, 2020). This condition poses a significant threat to patient lives, with the recurrent nature of epileptic seizure exerting a persistent negative impact, both physically and psychologically. Therefore, the diagnosis and treatment of epilepsy hold paramount significance.

Electroencephalography (EEG) refers to the recording of electrical activity generated by brain neurons on the scalp surface, widely attributed to synaptic activity in the cerebral cortex (Islam et al., 2023). EEG serves as a cost-effective, intuitive, and convenient method for recording brain activity by affixing electrode patches to the patient’s scalp surface, facilitating the capture of EEG signals (Al-Hajjar and Al-Qurabat, 2023). Presently, the primary approach to epilepsy detection involves diagnosis by experienced specialist physicians through EEG observation. However, this method heavily relies on the expertise and proficiency of the specialist, potentially leading to misdiagnosis due to prolonged and intricate evaluations (Zanetti et al., 2020). Seizure manifest in various EEG signal patterns, with fluctuations observed even within the EEG signals of the same patient across different seizure onset times (Ingolfsson et al., 2024). Hence, there is an urgent need for an intelligent diagnosis system which can detect epilepsy automatically and accurately. This system is also called Computer-Aided Diagnosis Systems (CADS), with AI-based CADS offering potential enhancements in diagnostic speed and accuracy (Malekzadeh et al., 2021; Mandhouj et al., 2021; Shankar et al., 2021).

The study of EEG signal has a history of several decades. New diagnostic techniques and classification algorithms emerge one after another. The automatic seizure detection system mainly classifies the interictal and ictal stages of EEG signals. It involves extracting important features from EEG signals using various digital signal processing techniques, and then classifying the extracted features based on statistical characteristics, Machine Learning (ML) or Deep Learning (DL) (Wang et al., 2025). Feature extraction plays a pivotal role in discerning the presence or absence of epilepsy from EEG signals, with the efficacy of features directly influencing classifier performance (Hu et al., 2021). However, the non-linear and variable characteristics of EEG signals, coupled with inevitable noise interferences during acquisition, present challenges to the accuracy of automated epilepsy detection (van Westrhenen et al., 2023).

In recent years, great progress has been made in the field of seizure detection, many scholars have explored and proposed a variety of non-linear signal analysis strategies to deal with the complex characteristics of epileptic EEG (Mostafa et al., 2024; Wei et al., 2021). As a function of time, the feature extraction of EEG signal can be realized directly in time domain or converted to frequency domain for further analysis. Currently, the features extracted from EEG signals are generally classified into four categories: Time-domain features, frequency-domain features, time-frequency-domain features and nonlinear features. In the time domain, some researchers have proposed statistical properties such as Standard Deviation (STD) and mean (Savadkoohi et al., 2020). In Shah et al. (2024), they extracted STD, mean, kurtosis, and skewness from EEG signals as characteristics. In the frequency domain, seizures can be detected by calculating the Power Spectral Density (PSD) of the EEG signal. Liu et al. (2023) segmented EEG PSD into periodic and non-periodic components, facilitating automatic epilepsy detection through parameterization of the PSD. In the time-frequency domain, wavelet-based feature extraction is widely used in the field of seizure research (Savadkoohi et al., 2020). By mapping the original signal to the time-frequency joint domain, the Wavelet Transform (WT) cannot only display the overall information of the signal, but also highlight the local information of the signal well (Boonyakitanont et al., 2020). Some researchers have successfully realized the detection of epileptic seizures by using WT. Yogarajan et al. (2023) applied Stationary Wavelet Transform (SWT) to the pre-filtered EEG signals and extracted a range of features from the decomposed sub-bands, including Hjorth parameters, Mean Absolute Value (MAV), STD, kurtosis, Root Mean Square (RMS), adjacent sub-band MAV ratio, activity, mobility, and complexity. To reduce model complexity, they employed the Binary Dragonfly Algorithm (BDFA) for feature selection, retaining only approximately 13% of the most informative features. Similarly, L. V. Tran et al. utilized Discrete Wavelet Transform (DWT) for multi-resolution analysis of EEG signals, focusing on sub-bands D3∼D5 and A5 after decomposition. They extracted statistical features such as maximum, mean, median, number of zero-crossings, STD, kurtosis and skewness from these sub-bands. Furthermore, Binary Particle Swarm Optimization (BPSO) was applied to reduce the feature dimensionality by 75% for subsequent classification tasks (Tran et al., 2022). Capturing nonlinear features in EEG signals is also a key task for researchers, and some of the widely used non-linear features include entropy (Malekzadeh et al., 2021), Fractal Dimension (FD), maximum Lyapunov exponent, hurst exponent, etc. As a core metric, entropy can directly quantify the degree of dynamic change within the system and effectively evaluate the complexity of the system. In the field of seizure detection, a series of entropy evaluators have shown significant application potential, among which approximate entropy, fuzzy entropy (Acharya et al., 2019), Shannon entropy and sample entropy are widely used in the analysis process. Tsipouras (2019) used spectral entropy, which is the Shannon entropy of the PSD of the EEG signal. According to the current literature review, most studies tend to integrate multiple types of features to comprehensively capture the connections between data, so as to improve the efficiency of classification algorithms (Pidvalnyi et al., 2025). Khan et al. (2020) introduced an automatic epilepsy detection method relying on a hybrid Local Binary Pattern (LBP)-WT algorithm. In this approach, LBP transforms signals, while WT decomposes them, extracting features including kurtosis, semi-variance, and Interquartile Range (IQR), followed by epilepsy seizure diagnosis via a linear discriminant analysis classifier. In another study, Pale et al. (2024) extracted the mean amplitude, approximate zero crossing, relative and absolute PSD, and line length of the EEG signal as features to help identify different EEG patterns.

In the process of automatic seizure detection, after feature extraction, the design of efficient and accurate discriminant feature classifier is another key link in the whole process. In recent decades, due to the rapid development of machine learning and deep learning, they have been widely used in many fields (Hamed et al., 2025; Wang et al., 2023). For example, Ameen et al. classified cardiovascular diseases based on Electrocardiography (ECG) and Phonocardiography (PCG) (Ameen et al., 2024), and Abdel Hady et al. predicted abdominal fat in their research (Abdel Hady et al., 2024) and some research on Parkinson’s disease (Eliwa and Abd El-Hafeez, 2025). Similarly, there are also many applications in epileptic seizure detection. K-Nearest Neighbor (KNN) and Support Vector Machine (SVM) are the most commonly used forms of ML (Supriya et al., 2020). Chen et al. (2020) first computed the signal strength at each EEG data point and applied Empirical Mode Decomposition (EMD) to decompose suspicious EEG segments into Intrinsic Mode Functions (IMFs). Singular Value Decomposition (SVD) was then used to extract representative features from the IMFs. These features were subsequently fed into two one-class SVMs, and the final epilepsy detection was determined by combining their outputs. In the study by Savadkoohi et al. (2020), KNN and SVM were used to classify the best features after *t*-test and Sequential Forward Floating Selection (SFFS) selection. Al-Hadeethi et al. (2020) introduced another two-phase classification method for seizure detection, utilizing covariance matrices in conjunction with adaptive boosting and Least Squares-SVM (LS-SVM). At present, many researchers have focused on the field of neural networks, especially their diverse variants, and are committed to exploring the potential and advantages of these models in classification tasks (Eliwa et al., 2023; Eliwa et al., 2024). Yu and Meng (2023) designed a model of Convolutional Neural Network (CNN) with an Attention Mechanism (AM), leveraging the multifrequency characteristics of EEG signals to map multilayer correlation information onto a network topology. Zhang et al. (2024) introduced a brain-inspired impulse neural network amalgamating Spiking Neural Network (SNN) models with data augmentation and adversarial strategies. Ein Shoka et al. proposed a novel approach that integrates chaos theory for secure EEG-based seizure detection (Ein Shoka et al., 2023). The EEG time-series signals were first transformed into two-dimensional spectrograms using the Teager-Kaiser Energy Operator (TKEO). To ensure data privacy in open network environments, the spectrograms were encrypted using the Arnold Transform (AT) and the chaotic baker mapping algorithm. Subsequently, they used transfer learning to transfer the learning results of multiple pre-trained CNN models on large-scale image datasets to the classification task of epileptic seizures, and achieved an accuracy of 86.11% on the CHB-MIT dataset. Zhang et al. (2023) initially employed a 64th order Butterworth bandpass filter to mitigate EEG signal noise, segmenting the complete EEG signal into multiple segments via a sliding window. Subsequently, they normalized the EEG signal using z-scores and trained EEG net, deep convolution network, and shallow convolution network models with the processed data. Although CNNs have been widely used in the field of seizure classification due to their powerful learning ability, their ability to capture temporal features is relatively limited when processing data rich in time series information such as EEG signals, which limits their performance in this field to a certain extent, resulting in unsatisfactory classification results in some cases. Studies in recent years have shown that Long Short-term Memory (LSTM) dominates the field of seizure research. Liu et al. (2020) proposed a new deep Convolutional-LSTM (C-LSTM) model for the first time to detect tumors and seizures. In the study by Hussain et al. (2021), they presented a DL architecture integrating CNN-LSTM which decomposes EEG signals into time-domain, frequency-domain, and time-frequency-domain features for binary and ternary classification. The highest accuracy of 99.27% was attained using time-frequency domain signals, while 96.64 and 94.71% accuracy were achieved with frequency domain and time domain signals, respectively. However, compared with LSTM, Gated Recurrent Unit (GRU) shows a more streamlined parameter configuration and a more concise structural architecture, which gives it superior modeling capabilities when dealing with complex data sequences, which has led to extraordinary progress in the field of automatic seizure detection. In Zhang et al. (2022), they used a Bidirectional GRU (BiGRU) to classify the relative energy extracted from the WT sub-bands as features, and finally achieved an average sensitivity of 93.89% and a specificity of 98.49% on the CHB-MIT dataset. To enhance performance in epileptic seizure detection, researchers have increasingly adopted innovative methodologies. For example, Lih et al. (2023) divided the collected EEG signals into segments every 5 s, calculated the Pearson correlation coefficient matrix for these segments, discarded the lower triangle, and only vectorized the upper triangle as input features. By incorporating a learnable positional encoding into the embedding vectors of these correlation coefficients, the transformer architecture was able to more effectively capture spatial relationships between EEG channels. Shoeibi et al. (2021) manually extracted 50 distinct features, including mean, variance, mode, peak slope, largest Lyapunov exponent, Shannon entropy, and approximate entropy. Prior to classification, features were ranked using the fisher score, and only those with higher scores were retained. Their classifier design embedded an Auto-Encoder (AE) within a CNN framework, where the AE component facilitated the extraction of more informative representations from the input signals. However, their evaluation relied on a single random train-test split (70% training, 30% testing), rendering the results sensitive to data partitioning. Takahashi et al. (2020) introduced an AE-CNN architecture with a novel “non-seizure but abnormal” class. A patient-specific AE layer was used to monitor EEG anomalies, combining the unsupervised learning capability of AEs with CNNs’ effectiveness in image-based classification. This approach significantly reduced the false positive rate. Srinivasan et al. (2023) proposed a hybrid deep learning model that combines a 3D Deep Convolutional Auto-Encoder (3D-DCAE) for automatic feature learning with a Bidirectional LSTM (BiLSTM) for temporal sequence modeling. This architecture improved both training efficiency and classification accuracy, underscoring the benefits of integrating temporal and spatial modeling in EEG analysis.

As discussed above, deep learning techniques have become powerful tools for addressing the complex signal classification challenges in epileptic seizure detection. However, several issues remain unsolved. EEG data are inherently highly imbalanced, which can bias classifiers toward the majority class. Moreover, most existing oversampling or under-sampling methods are applied in isolation, potentially distorting the distribution of the minority class. In addition, many deep models directly process raw or filtered EEG signals without leveraging informative time-frequency features that can capture both spectral and temporal dynamics. Importantly, limited research has explored the integration of autoencoders with temporal models to fully exploit the spatiotemporal dependencies of EEG signals. To address these limitations, this study proposes a novel hybrid model, termed Convolutional Sparse Autoencoder-Gated Recurrent Unit (CSAE-GRU). The model first constructs a CSAE to enhance the sparsity and discriminability of learned representations. The weights obtained during CSAE pretraining are transferred to the classification model to reduce training cost. The pretrained CSAE outputs are flattened and then fed into a GRU network with 64 hidden units to capture temporal patterns, followed by a fully connected layer and a softmax activation for binary seizure classification. The main contributions of this work are summarized as follows:

- We develop an effective two-stage data balancing strategy that combines cluster-centroid under-sampling and Borderline-Synthetic Minority Oversampling Technique (BLSMOTE) to address severe class imbalance inherent in seizure detection.
- We propose a novel CSAE–GRU classification framework that integrates handcrafted feature extraction with sparse representation learning and temporal modeling, thereby reducing model complexity and improving computational efficiency.
- We employ transfer learning to reuse the pretrained sparse encoder’s parameters, reducing training time and enhancing generalization across different EEG datasets.

The sections of this paper are organized as follows. Section 2 outlines the two datasets utilized in the study, alongside a comprehensive description of the proposed methodology. Section 3 presents the experimental results and discussion of this study. The conclusion of the paper is provided in section 4. Section 5 discusses the limitations of this study and outlines directions for future research.

## 2 Materials and methods

### 2.1 Datasets

The data used in this study were obtained from publicly available datasets, including the Bonn dataset and the CHB-MIT epilepsy database, both of which are widely utilized in epilepsy research. All data adhere to applicable ethical standards and were fully anonymized at the time of collection, containing no information that could identify individual participants. This study strictly complies with the ethical principles outlined in the declaration of Helsinki, and the datasets were exclusively used for scientific research purposes.

#### 2.1.1 Bonn dataset

**FIGURE 1:**
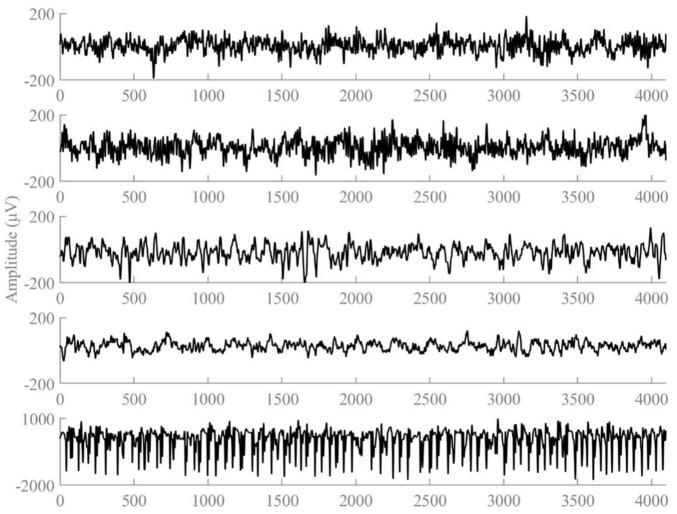
Examples of the five types of EEG signals.

The first dataset is a publicly available dataset provided by Dr. R.G. Andrzejak, University of Bonn, Germany. The dataset was recorded by the same 12-bit analog-to-digital converter, and consisted of five subsets (A, B, C, D, and E). Dataset A is the EEG signals obtained from five healthy volunteers in the awake and eyes-open state, dataset B is the EEG signals obtained from five healthy volunteers in the awake and eyes-closed state, datasets C∼E are from the EEG archives of five different patients with preoperative diagnosis. Datasets C, D correspond to interictal states, while dataset E represents the ictal state. Each of these five types of datasets consists of 100 segments, and the sampling frequency of each segment is 173.61 Hz, with the duration time of 23.6 s. Each fragment contains a total of 4,097 sample points. Figure 1 plots the EEG signal of the 1st segment of each subset. We employed 10 distinct classification tasks of the Bonn dataset, the specifics of which are delineated in Table 1.

**TABLE 1:** Classification tasks on the Bonn dataset.

Cases	Classification tasks	Description
Case 1	A VS. E	Healthy-Seizure
Case 2	B VS. E	Healthy-Seizure
Case 3	C VS. E	Interictal-Seizure
Case 4	D VS. E	Interictal-Seizure
Case 5	AB VS. E	Healthy-Seizure
Case 6	CD VS. E	Interictal-Seizure
Case 7	BC VS. E	No seizure-Seizure
Case 8	ABCD VS. E	No seizure-Seizure
Case 9	A VS. D	Healthy-Interictal
Case 10	AB VS. CD	Healthy-Interictal

#### 2.1.2 CHB-MIT dataset

The second dataset used in this study is the CHB-MIT scalp EEG database, which was created by the Massachusetts Institute of Technology in collaboration with Boston Children’s Hospital. The EEG signals were recorded at a sampling rate of 256 Hz and a resolution of 16 bits. The dataset records long-term EEGs of 5 males aged 3–22 years and 17 females aged 1.5–19 years (Ru et al., 2024). The EEG recordings of each patient are stored in a separate file in the European data format, and each file usually contains at least 1 h of continuous data, totaling about 976 h. A total of 185 epileptic seizure events were recorded in the entire dataset. All epileptic seizure events were annotated by professionals in the “summary” file, providing the start and end time of the file and the precise start and end time of each epileptic seizure. It is worth noting that the occurrence of epileptic seizures showed significant differences between different patients. Following the method proposed by Yang et al. (2021) and considering that frequent seizures would reduce the practical significance of the epileptic seizure detection task, this study focused on patients with less than 10 seizures. Therefore, EEG recordings from 10 selected patients were used in this experiment. The fundamental information of the 10 patients in the CHB-MIT dataset utilized in this experiment is presented in Table 2. We extracted ictal EEG segments from the annotated seizure onset to the seizure offset and applied a 2-s sliding window with 50% overlap (step size of 1 s) to generate multiple fixed-length seizure samples. To ensure a clear distinction between ictal and interictal periods, we defined the EEG signal at least 4 h away from the seizure event as the interictal period (Dissanayake et al., 2021). This time interval was chosen to ensure that the interictal data reflect the patient’s stable baseline brain activity, free from the transient physiological changes that occur before or after a seizure. These interictal signals were then segmented into non-overlapping 2-s epochs.

**TABLE 2:** Basic patient information on the CHB-MIT dataset.

Number	Sex	Age	Number of channels	Number of seizures	Seizure duration (s)	Recording duration (h)
Pt01	F	11	23	7	442	40.55
Pt 03	F	14	23	7	402	38
Pt 05	F	7	23	5	558	39
Pt 09	F	10	23–24	4	276	67.87
Pt 10	M	3	23	7	447	50.02
Pt 14	F	9	23	8	169	26
Pt 19	F	19	23	3	236	28.93
Pt 20	F	6	23	8	294	27.6
Pt 21	F	13	23	4	199	32.83
Pt 23	F	6	23	7	424	26.56
Total	–	–	–	60	3,447	377.36

### 2.2 Methods

**FIGURE 2:**
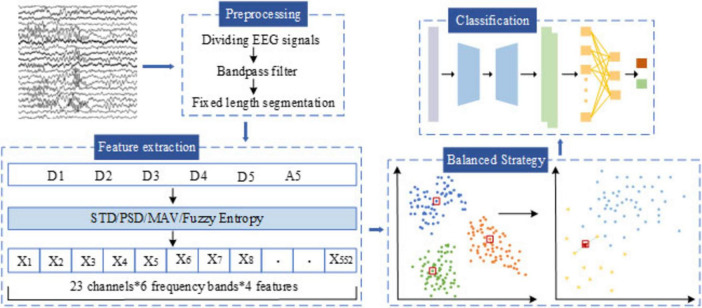
General structure of the proposed methodology.

The overall workflow of the proposed automatic epileptic seizure detection system is illustrated in Figure 2. First, EEG signals are preprocessed through bandpass filtering, followed by multi-scale feature extraction using DWT. Next, a tailored data balancing strategy is employed to address the severe class imbalance between ictal and interictal samples. Finally, a hybrid deep learning classification model, combining CSAE and GRU, is introduced to enable accurate seizure detection.

#### 2.2.1 Data preprocessing

The original EEG signals exhibit high dynamics and non-stationarity, and it is often subject to interference from physiological noise, environmental noise, and 50/60 Hz industrial frequency signals during acquisition. In order to ensure the accuracy and reliability of subsequent EEG signal analysis, it is imperative to remove the noise in the original EEG signals and enhance the signal-to-noise ratio.

Initially, we used the 5th-order Butterworth band-pass filter in the frequency range of 0.5∼60 Hz to filter the artifacts and noise from the original EEG signals, this configuration effectively removes low-frequency drift, baseline fluctuations, and certain power line interference (Shah et al., 2024). The Butterworth filter is an Infinite Impulse Response (IIR) filter characterized by an extremely flat frequency response within the passband. The transfer function of an *n*_*th* order Butterworth filter is shown in Equation 1.


$${\left|H\,\left(j\,\omega \right)\right|}^{2}=\frac{1}{1+{\left(\omega /\omega_{c}\right)}^{2\,n}}$$ (1)

where ω_*c*_ is the cutoff frequency and *n* is the filter order controlling the roll-off rate.

To further capture the time-frequency characteristics of the EEG signals, WT was applied in the experiment. It is widely employed in signal processing, facilitates signal decomposition into sub-signals of various scales, thereby capturing local features of the signal across different frequencies. This transform utilizes a window function of variable size to decompose a signal. For a signal *x*(*t*), the expression for Continuous Wavelet Transform (CWT) is shown in Equation 2.


$$C\,W\,T\,\left(a,b\right)=\int_{-\infty }^{\infty }x\,\left(t\right)\,\frac{1}{\sqrt{\left|a\right|}}\,\psi^{*}\,\left(\frac{t-b}{a}\right)\,dt$$ (2)

where, ψ is a wavelet function, ψ*denotes the conjugate complex of the wavelet function, a and b denote the scale and displacement parameters, respectively. CWT poses a significant computational challenge due to the necessity of determining the wavelet transform function at all scales. DWT offers an efficient solution to this issue by converting the scaling and displacement parameters into exponential powers of DWT is shown in Equation 3.


$$D\,W\,T\,\left(j,k\right)=\frac{1}{\sqrt{\left|2^{j}\right|}}\,\sum x\,\left(t\right)\,\psi^{*}\,\left(\frac{t-k\,2^{j}}{2^{j}}\right)$$ (3)

**FIGURE 3:**
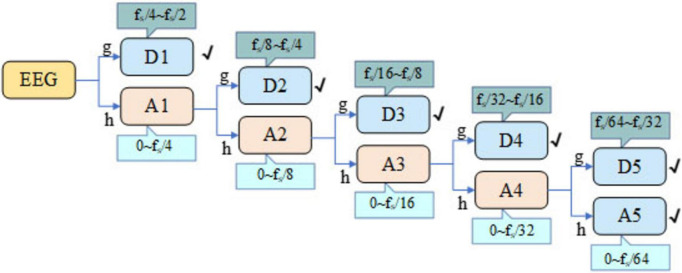
DWT five-level decomposition schematic.

If the signal is divided from the delta to the gamma band by the traditional method, it cannot adapt to the characteristics of EEG because of the fixed frequency band. Compared with this classification, the multi-scale and flexible analysis of DWT can observe the changes of EEG signals at different scales. In addition, DWT can effectively remove the noise in EEG signal and provide accurate information in time-domain and frequency-domain. The DWT employed the Daubechies 4 (db4) wavelet because of its compact support, orthogonality, and smooth waveform, which make it highly suitable for capturing the transient and non-stationary characteristics of EEG signals. Its four vanishing moments enable precise reconstruction of the underlying EEG rhythms while minimizing boundary distortion during multi-level decomposition. In the first-stage decomposition, the signal is processed by the DWT, which successively applies low-pass and high-pass filters followed by down-sampling. In accordance with Nyquist’s theorem, the original EEG signal is decomposed at the first level to yield two sub-bands: Approximation (A1) and Detail (D1), spanning the frequency ranges of 0 to f_*s*_/4 and f_*s*_ /4 to f_*s*_ /2, respectively. Likewise, further decomposition operations are performed on A1, dividing it into the higher-frequency component D2 and lower-frequency component A2. Subsequent iterations of decomposition entail further division of the approximation coefficients through the application of low-pass and high-pass filters, alongside down-sampling. The schematic diagram illustrating the five-level discrete wavelet transform of the EEG signal is depicted in Figure 3. Through five-level DWT, the EEG signals are converted into sub-bands A5, D5, D4, D3, D2, and D1, the corresponding frequency ranges of the Bonn dataset are 0∼2.713, 2.713∼5.425, 5.425∼10.85, 10.85∼21.7, 21.7∼43.4, and 43.4∼86.8 Hz, respectively, and the corresponding frequency ranges of the CHB-MIT dataset are 0∼4, 4∼8, 8∼16, 16∼32, 32∼64, and 64∼128 Hz, respectively.

#### 2.2.2 Feature extraction

In this section, we conduct feature extraction for each sub-band obtained after the DWT for seizure detection. In this study, we focus on extracting four features from each of the six sub-bands, emphasizing time-frequency-domain and nonlinear features. These features include: MAV, STD, PSD, and Fuzzy Entropy (FuEn). The extracted features are summarized in Table 3. For the Bonn dataset, which contains single-channel EEG recordings, the resulting feature matrix has a size of *N × 24* (*N* denotes the number of EEG segments). The CHB-MIT dataset includes 23 EEG channels, so its feature matrix has a size of *N × 552*. Each row represents an EEG segment, and each column corresponds to an extracted feature. A total of 80% of the feature samples are allocated to the training set, and the remaining 20% to the test set. The training set is balanced through a two-stage procedure described in the following section, while the test set retains the original class distribution to better reflect the data ratio encountered in clinical practice.

**TABLE 3:** Detailed description of dataset features.

Datasets	Sub-band	Extracted features	Number of channels	Features per segment	Feature matrix	Train/test ratio
Bonn	A5, D1∼D5	MAV, STD, PSD, FuEn	1	24	N × 24	0.8/0.2
CHB-MIT	A5, D1∼D5	MAV, STD, PSD, FuEn	23	552	N × 552	0.8/0.2

MAV, known for its simplicity and effectiveness, offers notable advantages in capturing abnormal amplitude variations in EEG signals of epilepsy patients. The mathematical expression for the MAV is shown in Equation 4.


$$M\,A\,V=\frac{1}{N}\,\sum_{i=1}^{N}\left|x\,\left(i\right)\right|$$ (4)

where, *N* represents the number of samples in the EEG signal, and *x*(*i*) denotes the amplitude value of the *i^th^* sample.

STD, a crucial statistical measure, is primarily employed to assess the volatility and instability of a signal. It quantifies the degree of dispersion of signal values relative to the mean signal value. When abnormal data points exist within the signal, these outliers typically result in an elevated STD value. Consequently, monitoring changes in standard deviation enables effective capture of EEG dynamics to ascertain seizure occurrences. The mathematical expression for the STD is shown in Equation 5.


$$S\,T\,D=\sqrt{\frac{1}{N}\,\sum_{i=1}^{N}{\left(x_{i}-\bar{x}\right)}^{2}}$$ (5)

where, *N* denotes the number of samples in the signal, and *x_i_* signifies the value of the ı*^th^* sample.

PSD of EEG signals serves as a crucial tool for analyzing changes in the time and frequency domains of brain activity. The EEG signal consists of multiple frequency components, including δ-waves, θ-waves, α-waves, β-waves, and γ-waves. Abnormal spectral characteristics are frequently associated with pathological states. Thus, by comparing the energy spectral density of patient groups with that of normal groups, we can more accurately identify and diagnose brain disorders, facilitating improved screening of seizure and inter-ictal periods in epilepsy.

Entropy, serving as a characterization metric, is commonly utilized to depict the uncertainty, disorder, or information content of a system (Jindal et al., 2019). In time series analysis, entropy is introduced to describe the complexity and regularity of signals. Traditional entropy methods often encounter challenges when delineating boundaries between uncertain and definite classes. To address this issue, Lotfi Zaden introduced fuzzy theory (Fu et al., 2020). Fuzzy entropy, as an extension of traditional entropy, is particularly suitable for the analysis of non-stationary time series and biomedical signals.

#### 2.2.3 Two-stage data balancing strategy

Data class imbalance remains a critical challenge in EEG-based epileptic seizure detection, significantly impacting model performance. In real-world clinical datasets, the number of ictal samples is substantially lower than that of interictal samples. Even when overlapping sliding windows are used to augment data, severe imbalance persists. This pronounced disparity often leads to biased classification, causing classifiers to be insensitive to minority class data and thereby degrading performance in clinical applications. For instance, in the first patient of the CHB-MIT dataset, there are 25,200 interictal samples compared to only 435 ictal samples, resulting in a ratio of approximately 60:1. To address this substantial imbalance, we propose a two-stage data balancing strategy comprising interictal under-sampling and ictal oversampling. The detailed implementation of these stages is described below.

##### 2.2.3.1 Under-sampling stage based on cluster centroids

We first applied a cluster centroid-based under-sampling method to reduce the number of interictal samples. This technique leverages k-means clustering to group the interictal data, where the number of clusters corresponds to the target number of retained samples. In this study, the sampling strategy parameter was set to 0.5, ensuring a 2:1 ratio between interictal and ictal samples. To maintain experimental reproducibility, the random seed was fixed at 42. Within each cluster, synthetic representative samples were generated by computing the centroid of the data points, effectively reducing the volume of interictal data while preserving essential information.

##### 2.2.3.2 Oversampling stage based on the BLSMOTE

Following the under-sampling process, although the class distribution was substantially improved, the number of seizure samples remained insufficient for ideal classification performance. To address this, we employed the BLSMOTE technique to further augment the seizure class and achieve complete class balance. Unlike traditional SMOTE, which performs indiscriminate random interpolation across all minority samples, BLSMOTE focuses on generating synthetic samples near the decision boundary. This targeted oversampling helps avoid redundancy and reduces the risk of introducing noise from distant or non-informative regions. Specifically, BLSMOTE first identifies minority class samples located near the decision boundary and then uses the KNN algorithm to interpolate between these boundary samples and their neighboring seizure instances. In our experiment, the BLSMOTE sampling strategy was set to 1, and the random seed was fixed at 42 to ensure reproducibility. The generated synthetic seizure samples were then incorporated into the training set, with care taken to prevent data leakage. In this study, BLSMOTE was applied in the feature space rather than directly on raw EEG segments. Synthesizing in the feature domain preserves the physiological interpretability of the data and avoids potential distortions that may arise when manipulating raw EEG signals. In contrast, oversampling in the time-domain would require additional preprocessing steps such as filtering, segmentation, and feature extraction, thereby increasing computational overhead. By operating in the feature space, our method enhances computational efficiency while maintaining the integrity and biological validity of the dataset.

#### 2.2.4 Classification

**FIGURE 4:**
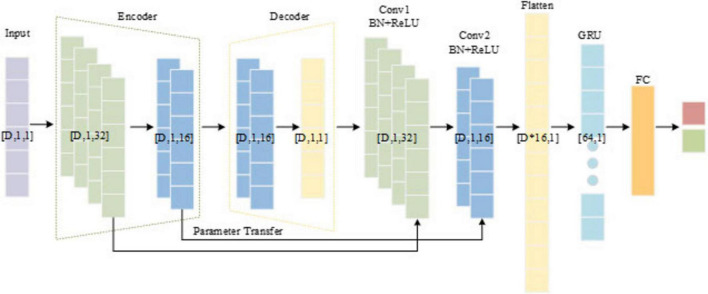
The structure of the proposed CSAE-GRU.

In this study, a hybrid model named CSAE-GRU is developed to achieve reliable epileptic seizure detection by combining convolutional sparse feature learning with temporal sequence modeling. The overall structure is shown in Figure 4. The model begins with a CSAE that is responsible for learning compact and informative feature representations. The input to the CSAE consists of the handcrafted features. Before entering the network, these features are normalized to the range [0, 1] and reshaped into a four-dimensional tensor of size D* 1 * 1 * N, where D denotes the feature dimension and N the batch size. The encoder is designed to capture the local dependencies among neighboring features through convolutional operations, progressively increasing the number of channels from 1 to 32 and then reducing it to 16. Each convolutional block includes batch normalization, a ReLU activation, and dropout regularization to enhance sparsity and prevent overfitting. The decoder mirrors the encoder structure, reconstructing the original feature maps from the latent representation by gradually restoring the channel dimensions from 16 back to 1. The reconstruction process is guided by a loss function, as shown in Equation 6.


$$L={||x-\hat{x}||}_{2}^{2}+\lambda \,{||h||}_{1}$$ (6)

where *x* represents the original input signal, $\hat{x}$ represents the reconstructed signal output by the decoder, *h* is the encoded feature, and λ controls the sparsity strength.

After the CSAE pre-training phase, the encoder weights used to capture key feature patterns are transferred to two convolutional blocks. This parameter transfer preserves the learned spatial structure and accelerates the convergence of the classifier. The output of the convolutional blocks is flattened into a one-dimensional vector and passed to a GRU network consisting 64 hidden units. The GRU module plays a central role in capturing the temporal evolution and contextual dependencies among the reconstructed EEG feature sequences. Each GRU cell consists of an update gate and a reset gate that regulate the flow of information through the network. The update gate controls how much of the past information is retained, while the reset gate determines how much of the previous state should be forgotten when processing a new input. This gating mechanism allows the GRU to effectively model long-term dependencies and mitigate the vanishing-gradient problem, which is often encountered in traditional recurrent neural networks. By processing feature vectors, the GRU captures transitions between interictal and ictal states, thus enhancing the model’s ability to distinguish seizure patterns. The final hidden state of the GRU is fed into a fully connected layer followed by a softmax activation function to produce the binary classification output.

The CSAE and GRU components are trained using the Adam optimizer with an initial learning rate of 0.001. The CSAE is trained for a maximum of 30 epochs with a reconstruction-loss threshold as an early-stopping criterion, while the GRU-based classifier is trained for up to 50 epochs. Dropout rates of 0.2 in the autoencoder and 0.5 in the classifier are used to further mitigate overfitting. The architecture and hyperparameters are kept consistent across all experiments on both datasets to maintain fair comparisons. The detailed layer configuration of the proposed CSAE-GRU network is presented in Table 4. The experimental parameters and training settings are summarized in Table 5 to ensure the reproducibility of the proposed framework.

**TABLE 4:** Detailed layer configuration of the proposed CSAE-GRU model.

Module	Layer	Parameters	Activation/ regularization
CSAE encoder	Conv1	3 × 1 kernel, 32 filters	BN-ReLU-Dropout (0.2)
	Conv2	3 × 1 kernel, 16 filters	BN-ReLU-Dropout (0.2)
CSAE decoder	Conv1	3 × 1 kernel, 16 filters	BN-ReLU
	Conv2	3 × 1 kernel, 1 filter	Regression layer
Transferred layers	Frozen Conv1	3 × 1 kernel, 32 filters	BN-ReLU
	Frozen Conv2	3 × 1 kernel, 16 filters	BN-ReLU
GRU module	GRU	Hidden units: 64	Dropout (0.5)
Classifier	Fully connected	Units: 2	Softmax

**TABLE 5:** Experimental setup and training parameters.

Parameter	Value	Description
Optimizer	Adam	Adaptive momentum adjustment
Learning rate	0.001	Empirically determined for stable convergence
Batch size	32	Balances training stability
Epochs	30/50	Maximum training iterations for each stage
Dropout rate	0.2/0.5	Prevents overfitting and improves generalization

**FIGURE 5:**
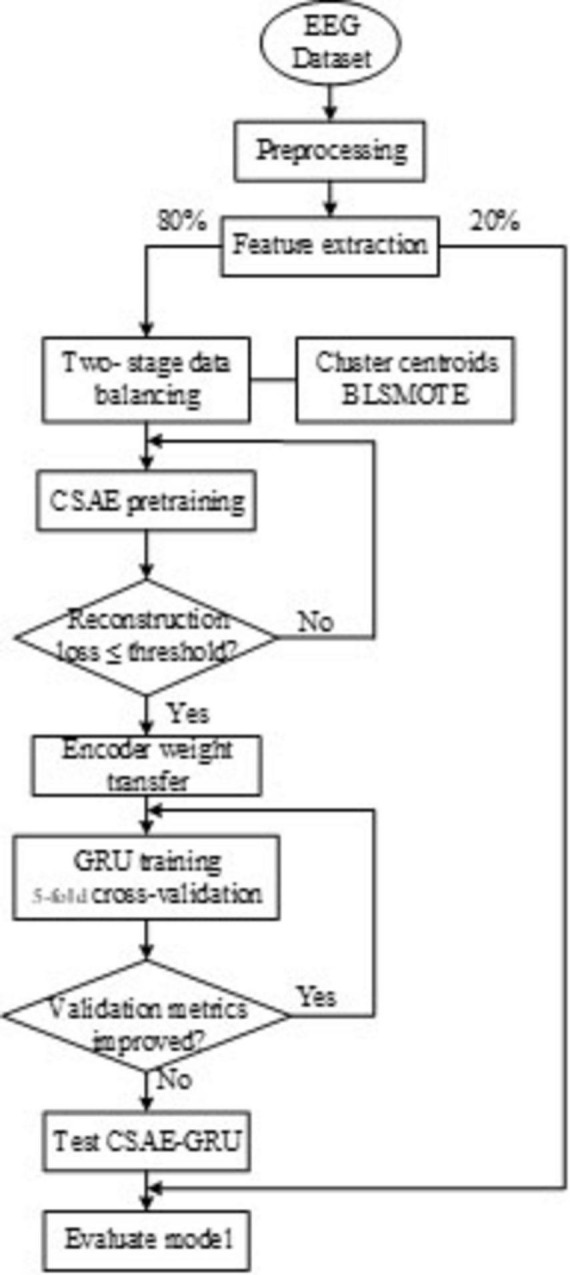
Flowchart of the proposed CSAE-GRU algorithm.

The proposed CSAE-GRU model aims to achieve accurate and stable epileptic seizure detection through a combination of sparse feature reconstruction and temporal pattern learning. In this framework, a CSAE is first used to reconstruct representative EEG features, and the learned encoder weights are then transferred to the subsequent GRU-based classifier. This design allows the model to retain discriminative information learned during unsupervised pretraining while improving the generalization ability of the classifier. Algorithm 1 outlines the entire procedure of the proposed framework, and Figure 5 presents the flowchart, including the decision steps for reconstruction loss and validation accuracy during training.

Algorithm 1 Pseudocode of the proposed epileptic seizure detection framework.

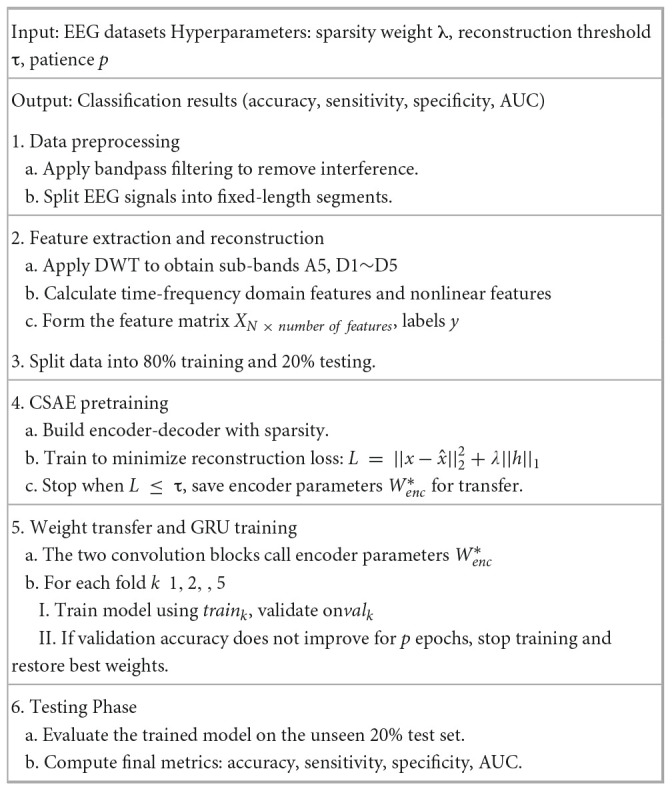

## 3 Results and discussion

### 3.1 Evaluation indicators

In this study, we employed five essential metrics, namely accuracy, sensitivity, specificity, precision and F1 score to evaluate the performance of the proposed automatic seizure diagnostic method. These indicators are calculated as shown in Equations 7–11.


$$A\,c\,c\,u\,r\,a\,c\,y\,\left(A\,C\,C\right)=\frac{T\,P+T\,N}{T\,P+T\,N+F\,P+F\,N}$$ (7)

$$\mathrm{Sensitivity}\,\left(\mathrm{SEN}\right)=\frac{T\,P}{T\,P+F\,N}$$ (8)

$$\mathrm{Specificity}\,\left(S\,P\,E\right)=\frac{T\,N}{T\,N+F\,P}$$ (9)

$$\mathrm{Precision}\,\left(P\,R\,E\right)=\frac{T\,P}{T\,P+F\,P}$$ (10)

$$\mathrm{F1}\,\mathrm{score}\,\left(F\,1\right)=\frac{2\,T\,P}{2\,T\,P+F\,N+F\,P}$$ (11)

where, True Positive (TP), False Positive (FP), True Negative (TN) and False Negative (FN) denote the four classification outcomes, reflecting the model’s ability to correctly or incorrectly identify epileptic and non-epileptic samples.

### 3.2 k-fold cross-validation

In view of the fact that the dataset used in the experiment was composed of EEG signals recorded by patients under uniform conditions, and there was no clear topic or category boundary set in advance, we used the k-fold cross-validation method to ensure the generality of the test through multiple iterations of training and verification, and to reduce the possible bias caused by random division of data. In this paper, the performance of the model is evaluated using the fivefold cross-validation method. In this approach, all data are randomly divided into 5 mutually exclusive subsets. During each iteration, four subsets are randomly selected for model training, while the remaining subset is reserved for testing. This process is repeated five times, with each subset being used as the test set exactly once. Subsequently, the average performance across all iterations is calculated to assess the overall model performance.

### 3.3 Results

#### 3.3.1 Distribution of features

**FIGURE 6:**
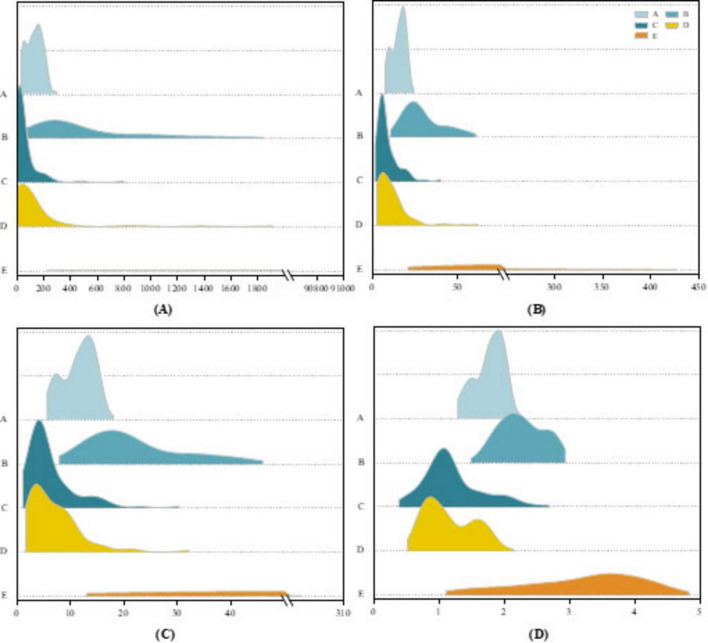
Ridgeline plot on the Bonn dataset for **(A)** PSD features, **(B)** STD features, **(C)** MAV features, **(D)** Fuzzy entropy features.

To evaluate the discriminative power of the extracted features, we visualize them for interpretability. Figure 6 illustrates the distributions of four representative features extracted from the D3 sub-band across five EEG signal types in the Bonn dataset. As shown in Figures 6A–C, horizontal breakpoints are introduced to distinguish the categories. The ictal (type E) signals exhibit markedly higher values across three features compared to the other four types. In particular, the PSD reaches nearly 91,000, reflecting the dramatic energy fluctuations characteristic of seizure activity. The STD values for healthy signals (types A and B) and interictal signals (types C and D) remain consistently low with narrow value ranges, whereas the STD for ictal signals displays considerable variability. The MAV exhibits a similar trend to PSD, with ictal signals reaching peak values exceeding 300. Figure 6D presents the distribution of fuzzy entropy across the five signal categories. A clear hierarchical structure emerges, where the first four signal types exhibit relatively low fuzzy entropy values. Notably, type D signals show the lowest entropy, while type E signals demonstrate significantly higher fuzzy entropy, indicating increased signal complexity and disorder during seizure events.

**FIGURE 7:**
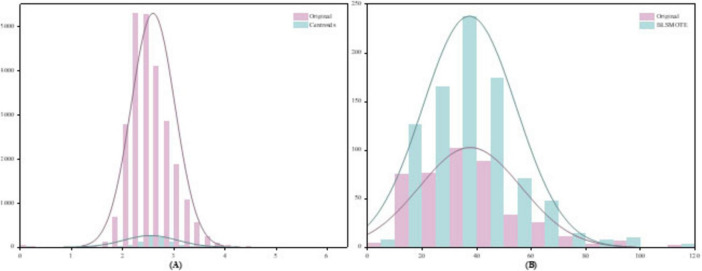
Results of the two-stage data balancing strategy **(A)** Under-sampling of interictal samples using cluster centroids, **(B)** Oversampling of ictal samples using BLSMOTE.

To validate the effectiveness of the proposed two-stage data balancing strategy, we present a case study using data from the first patient in the CHB-MIT dataset. Figure 7 illustrates the feature distribution of EEG samples after under-sampling and oversampling. Specifically, Figure 7A shows the distribution of interictal samples following cluster centroid under-sampling, while Figure 7B demonstrates the distributional changes after applying BLSMOTE-based oversampling. To compare the distributional shifts between ictal and interictal samples before and after balancing, both probability density estimation curves and histograms are employed. In Figure 7A, the original interictal sample distribution exhibits pronounced skewness and high concentration in specific regions. After applying k means-based cluster centroid under-sampling, the resulting distribution displays a significant reduction in redundant samples while preserving the core morphological characteristics of the original feature space. Compared to conventional random under-sampling, this method better retains feature diversity and minimizes information loss. As shown in Figure 7B, the BLSMOTE-generated synthetic samples exhibit a broader and more dispersed distribution across the feature space. Unlike standard SMOTE, which performs simple linear interpolation, BLSMOTE focuses on generating samples in ambiguous boundary regions, thus capturing more discriminative information. This enhancement allows the classifier to more effectively distinguish minority class instances and improves overall model robustness in imbalanced data scenarios.

**FIGURE 8:**
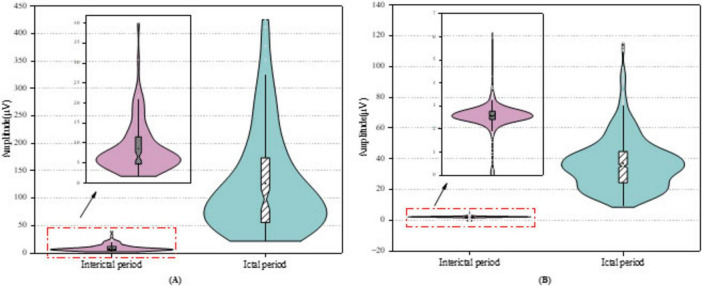
Violin plots of feature distribution on the datasets for **(A)** Bonn dataset, **(B)** CHB-MIT dataset.

Figure 8 presents violin plots of the STD feature extracted from the D3 sub-band of the Bonn dataset Figure 8A and the CHB-MIT dataset Figure 8B. To minimize visual clutter and avoid confusion from multiple features, we selectively display only one representative sub-band and feature. As shown in the plots, the STD values of ictal signals are significantly higher than those of interictal signals in both datasets. The Bonn dataset exhibits considerable signal variability, with ictal STD values reaching up to approximately 400 μV, whereas in the CHB-MIT dataset, the maximum ictal value is around 120 μV. This discrepancy may stem from individual differences among patients or underlying pathological variations between datasets. Additionally, ictal signals not only exhibit elevated feature values but also display a noticeable long-tail distribution with extreme outliers. In contrast, interictal signals are characterized by lower and more stable STD values, with fluctuations confined to a narrower range. This stark difference in distribution further highlights the potential of STD as a discriminative feature for seizure detection.

#### 3.3.2 Experiment results

The experiments were conducted on a computer equipped with an Intel Core i7-13700KF processor (3.4 GHz), 32 GB RAM, and a 64-bit Windows 11 Professional operating system. All implementations were performed using MATLAB R2022a with its deep learning and signal processing toolboxes. The computational cost of the proposed framework depends primarily on the number of training epochs and feature processing steps. To quantitatively assess the computational efficiency of the proposed framework, both execution time and memory utilization were continuously monitored during training and testing. For the CHB-MIT dataset, the average runtime per fold was 58.69 s, yielding a total runtime of 300.83 s across fivefold. For the Bonn dataset, the average runtime per fold was 1.21 and 6.17 s overall. The MATLAB memory consumption remained stable at approximately 8.2–8.5 GB throughout all experiments.

**FIGURE 9:**
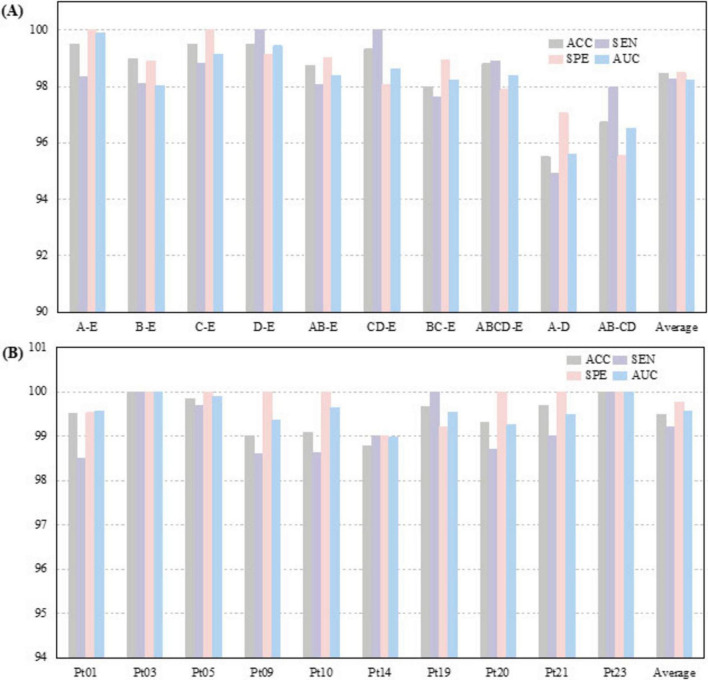
Classification results of the proposed CSAE-GRU classifier on the datasets for **(A)** Bonn dataset, **(B)** CHB-MIT dataset.

The proposed CSAE-GRU model was comprehensively evaluated on both the Bonn and CHB-MIT datasets. The detailed results are presented in Figure 9 and Tables 6, 7. In terms of overall average performance, on the Bonn dataset, the CSAE-GRU model achieved an average performance of 98.46% ACC, 98.27% SEN, 98.50% SPE, and 98.23% AUC. On the CHB-MIT dataset, the model demonstrated even higher performance, with respective values of 99.49, 99.21, 99.77, and 99.57%. The CSAE-GRU model achieved 100% SPE in some scenarios. For example, in the A-E and C-E scenarios of the Bonn dataset, and in the Pt03 and Pt23 of CHB-MIT, the model can accurately distinguish non-epileptic EEG signals, which is a critical point in actual clinical applications. Similarly, the model achieved 100% SEN in tasks such as D-E and CD-E in the Bonn dataset, as well as in CHB-MIT subjects Pt03, Pt19, and Pt23, indicating zero missed detections in these cases. This result highlights the model’s strong potential to reliably capture seizure-related patterns, which is of great significance for applications requiring high sensitivity. Horizontal comparisons across different task configurations reveal that in more complex A-D and AB-CD combinations, the boundaries between the two types of signals are relatively fuzzy. The model’s classification ACC slightly declined but remained above 95%. This demonstrates the model’s ability to capture subtle discriminative features through CSAE, even under complex scenarios. In the CHB-MIT dataset, although the overall performance was consistently high, a slight performance drop was observed for subject Pt14. The observed results indicate that inter-subject variability, such as differences in seizure onset zones, clinical presentations, and EEG recording configurations, poses a significant challenge to model generalization and performance. These observations underscore the need for further optimization to enhance the model’s adaptability and robustness across diverse patient populations.

**TABLE 6:** Specific results of the proposed model on the Bonn dataset.

Case	ACC (%)	SEN (%)	SPE (%)	PRE (%)	F1 (%)	AUC (%)
A–E	99.50	98.33	100	99.16	98.75	99.89
B–E	99.00	98.11	98.92	98.47	98.29	98.04
C–E	99.50	98.82	100	99.41	99.11	99.15
D–E	99.50	100	99.13	99.56	99.78	99.44
AB–E	98.75	98.06	99.03	98.54	98.30	98.40
CD–E	99.33	100	98.06	99.03	99.51	98.63
BC–E	98.00	97.62	98.95	98.29	97.95	98.24
ABCD–E	98.80	98.90	97.89	98.39	98.65	98.39
A–D	95.50	94.92	97.07	96.00	95.45	95.59
AB–CD	96.75	97.98	95.54	96.76	97.37	96.52

**TABLE 7:** Specific results of the proposed model on the CHB-MIT dataset.

Case	ACC (%)	SEN (%)	SPE (%)	PRE (%)	F1 (%)	AUC (%)
Pt01	99.52	98.51	99.53	99.02	98.76	99.56
Pt03	100	100	100	100	100	100
Pt05	99.85	99.69	100	99.85	99.77	99.89
Pt09	99.00	98.59	100	99.29	98.94	99.37
Pt10	99.09	98.63	100	99.31	98.97	99.65
Pt14	98.79	99.00	99.00	99.00	99.00	98.97
Pt19	99.67	100	99.20	99.60	99.80	99.55
Pt20	99.31	98.70	100	99.35	99.02	99.25
Pt21	99.69	99.00	100	99.50	99.25	99.50
Pt23	100	100	100	100	100	100

#### 3.3.3 Verification of the effectiveness of the CSAE-GRU model

To further verify the contribution of each component in the proposed CSAE-GRU architecture, we compared it with four additional variants: CNN, GRU, CNN-GRU, and CSAE-MLP. The results of all metrics are summarized in Table 8. As shown, the proposed CSAE-GRU consistently achieves the best performance across all evaluation indicators. Compared with the CNN model, CSAE-GRU improves ACC by 1.68 and 2.94% on the Bonn and CHB-MIT datasets, respectively, while the SEN increases by more than 3 percentage points. When compared with CNN-GRU, it is evident that replacing the conventional CNN with the CSAE module leads to further performance gains in all metrics, highlighting the noise-robustness advantage of CSAE-based feature learning. Unlike traditional CNNs, which tend to capture redundant or noisy spatial patterns, the convolutional sparse autoencoder enforces sparsity constraints and self-reconstruction objectives, thereby producing more discriminative and noise-resistant representations. To evaluate the contribution of temporal modeling, the GRU layer was replaced with a Multilayer Perceptron (MLP) while keeping the same CSAE encoder. On the CHB-MIT dataset, this modification resulted in decreases in several metrics, with accuracy dropping by 1.67% and specificity by 1.91%, among others. These results confirm that temporal dependency modeling is essential, particularly for long, multi-channel EEG sequences such as CHB-MIT. The GRU effectively leverages the structured spatial representations provided by CSAE to capture dynamic seizure-related transitions and suppress temporal noise, leading to superior overall performance.

**TABLE 8:** Ablation results of different model variants on the datasets.

Dataset	Model	ACC	SEN	SPE	PRE	F1
Bonn	CNN	96.80	95.06	96.84	95.95	95.50
	GRU	95.81	95.57	95.83	95.70	95.63
	CNN-GRU	97.20	96.30	97.26	96.78	96.54
	CSAE-MLP	97.35	97.87	97.34	97.61	97.74
	CSAE-GRU	98.48	98.27	98.50	98.36	98.31
CHB-MIT	CNN	96.55	94.44	96.64	95.54	94.99
	GRU	95.51	97.01	95.24	96.12	96.57
	CNN-GRU	98.81	98.15	98.83	98.49	98.32
	CSAE-MLP	97.82	97.46	97.86	97.66	97.56
	CSAE-GRU	99.49	99.21	99.77	99.49	99.35

**FIGURE 10:**
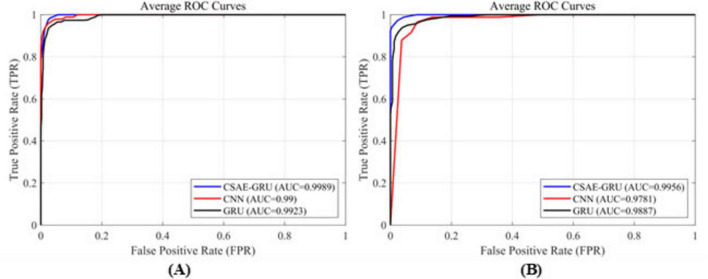
ROC curves for the three classification models **(A)** on the Bonn dataset, **(B)** on the CHB-MIT dataset.

In order to further provide an intuitive comparison of the performance among different classification models, Figure 10 presents the ROC curves of the proposed CSAE-GRU model in contrast to single CNN and GRU models. Figures 10A, B depict the average ROC curves obtained from fivefold cross-validation for the A-E classification task on the Bonn dataset and the Pt01 classification task on the CHB-MIT dataset, respectively. In both scenarios, the CSAE-GRU model achieved superior AUC values of 99.89 and 99.56%, respectively. Its ROC curves lie closer to the upper left corner, indicating excellent classification capability. Notably, when the False Positive Rate (FPR) was below 0.02, the True Positive Rate (TPR) remained above 99%, demonstrating the model’s ability to minimize missed detections without increasing false alarms. These results confirm that the CSAE-GRU model outperforms both standalone CNN and GRU models, further validating the effectiveness of combining CSAE for robust feature extraction with GRU networks for temporal sequence modeling. The integration of spatial sparsity and temporal dependency contributes to the model’s superior discriminative power in EEG-based seizure detection tasks.

### 3.4 Discussion

In order to evaluate the effectiveness of our proposed CSAE-GRU classification method compared to other methods, we have compared the method proposed in this paper with the methods proposed in the literature in recent years. Table 9 lists the results of a wide variety of methods proposed in recent years on the Bonn dataset compared to the method proposed in this paper. It is clear from the table that among all the proposed methods, our study achieves higher results. Raghu et al. (2019) and Al-Hadeethi et al. (2021) have extracted mathematical features of EEG signals based on linear algebra using matrices combined with algorithms to classify them, and they have achieved more than 95% of the classification results. Wang et al. (2023) proposed CNN-LSTM classification method which achieved more than 97.6% ACC in binary classification task, but the rest of the metrics are not mentioned. Recently in Liu et al. (2023)’s study also used PSD as features and added linear discriminant analysis on top of SVM and KNN. Ultimately, an ACC of 98.5% was achieved on D-E grouping, which is 1% lower compared to the ACC of our proposed method on D-E grouping. They reported an average computational time of 0.0111 s per 10-s EEG epoch. This value reflects the processing time for each individual segment rather than the total training or testing duration, and no information regarding memory utilization was provided. In contrast, the proposed CSAE-GRU model achieved an end-to-end runtime of 6.17 s on the Bonn dataset. Huang et al. (2024) extracted time-varying features from EEG signals using a Temporal Convolutional Neural Network (T-CNN), and then employed a Self-Attention (SA) Layer to weigh the importance of these features. The method achieved classification ACC of 97.37 and 93.5% on the A-E and B-E datasets, respectively. However, it is worth noting that the study only designed two classification experiments, limiting the validation of the method’s generalization capability. Mekruksavanich et al. (2024) enhanced sequential modeling by incorporating residual connections into the BiGRU architecture and further improved feature discriminability through the integration of the Convolutional Block Attention Module (CBAM). While their model achieved slightly higher SEN and SPE in the B-E classification task, it demonstrated inferior performance across the remaining groups compared to our proposed approach.

**TABLE 9:** Comparison of the previous methods and proposed method on Bonn dataset.

Papers	Year	Methods	Case [ACC (%)/SEN (%)/SPE (%)]
Mert and Akan (2018)	2018	EMD+IMF + PSD	A–E (97.90/100/95.76) B–E (83.70/78.38/76.66) C–E (96.40/97.88/94.11) D–E (93.00/97.88/88.55)
Raghu et al. (2019)	2019	Matrix determinant + SVM/KNN/MLP	B–E (96.06/98.45/93.65) C–E (97.60/95.75/99.45) D–E (97.62/94.90/99.45) AB–E (97.10/95.70/98.50) CD–E (96.95/97.60/94.30) ABCD–E (96.52/96.55/96.50)
Al-Hadeethi et al. (2021)	2021	KST +Mann-Whitney U +Covariance Matrix +AB-BP-NN	C–E (99.00/99.00/99.00) CD–E (98.00/98.00/97.00) ABCD–E (98.00/99.00/98.00)
Aayesha et al. (2022)	2022	Time domain + Spectrum + Non-linear features + LBP+ Feedforward Neural Network	A–E (96.67/93.33/100) B–E (91.67/93.33/90.00) C–E (91.67/90.00/93.33) D–E (85.00/83.33/86.67) AB–E (90.00/76.47/98.21) CD–E (91.11/91.18/91.07)
Liu et al. (2023)	2023	PSD + SVM/KNN/Decision tree (DT)/Linear Discriminant Analysis (LDA)	D–E (98.50/98.00/99.00) CD–E (98.70/97.00/99.50) ABCD–E (98.40/95.00/99.25)
Wang et al. (2023)	2023	Hybrid 3 CNN-1 LSTM	C–E (98.20/NA/NA) D–E (97.60/NA/NA) CD–E (97.90/NA/NA)
Huang et al. (2024)	2024	TCNN-SA	A–E (97.37/94.88/99.91) B–E (93.50/88.07/99.00)
Mekruksavanich et al. (2024)	2025	CNN+ ResBiGRU+ CBAM	A–E (99.00/99.00/99.00) B–E (99.00/99.00/99.00) C–E (97.50/97.50/97.50) D–E (98.50/98.50/98.50)
This work	2025	DWT + STD + PSD + MAV + Fuzzy entropy +CSAE-GRU	A–E (99.50/98.33/100) B–E (99.00/98.11/98.92) C–E (99.50/98.82/100) D–E (99.50/100/99.13) AB–E (98.75/98.06/99.03) CD–E (99.33/100/98.06) BC–E (98.00/97.62/98.95) ABCD–E (98.80/98.90/97.89) A–D (95.50/94.92/97.07) AB–CD (96.75/97.98/95.54)

Table 10 lists the compared results of our proposed method and other methods in recent years on the CHB-MIT dataset. Wei et al. (2019) designed an automatic seizure detection method based on the ability of CNNs to automatically extract and learn features, and designed a 12-layer CNN for classification. The CNN classification method that introduces MIDS and data enhancement has an increase in SEN and SPE compared with the original CNN classification method, but it is still lower than the method we proposed in this paper. Peng et al. (2021) also proposed a method for automatic diagnosis of epileptic seizure. Their EEG samples required approximately 0.26 s from preprocessing to outputting the class label. Unfortunately, the authors only reported the processing time for a single sample and did not specify the total execution time. Hu et al. (2021) achieved 98.97% ACC in his study using Deep Neural Network (DNN) for fusion of extracted features and then Hopfield Neural Network (HNN) for classification, however, only the accuracy of the proposed method was reported. We also refer to the study of Zhang et al. (2022) who first transformed the EEG signal using DWT, calculating the relative energy of specific wavelet bands as features, and then implemented automatic epilepsy detection using a BiGRU network, which achieved an ACC of 98.49% and a SEN of 93.89%. In addition, Tang et al. (2024) proposed a BiLSTM with an AM using Path signature to extract more representative features, achieving 99.09% ACC. They reported that the feature extraction process took 0.09–3.22 s, whereas the total execution time and memory utilization were not specified. Huang et al. (2025) introduced a novel framework Spatio-Term Feature Fusion with Dual Attention (STFFDA), employing CNN and BiLSTM networks to jointly capture spatial dependencies and temporal dynamics within raw EEG data. Comprehensive evaluation using 10-fold cross-validation on the CHB-MIT dataset, achieving both an ACC and SEN of 92.42%.

**TABLE 10:** Comparison of the previous methods and proposed method on CHB-MIT dataset.

Papers	Year	Methods	ACC (%)	SEN (%)	SPE (%)
Wei et al. (2019)	2019	CNN+ Raw data	NA	70.68	92.30
		k CNN+MIDS data		74.08	92.46
		CNN+ Data augmentation		72.11	95.89
Tian et al. (2019)	2019	FFT+WPD+PCA+CNN	98.30	96.70	99.10
Zanetti et al. (2020)	2020	Band power+5 Entropy +RF	NA	96.60	92.50
Peng et al. (2021)	2021	Stein kernel-based sparse representation	98.21	97.85	98.57
Li and Chen (2021)	2021	FFT+2D matrix+ SVM	98.47	98.28	98.50
Hu et al. (2021)	2021	MAS+MPSD+WPF+DNN+HNN	98.97	NA	NA
Zhang et al. (2022)	2022	DWT +Specific band energy +BiGRU	98.49	93.89	98.49
Tang et al. (2024)	2024	Path signature + BiLSTM+ AM	99.09	99.28	98.95
Shah et al. (2024)	2024	DWT+RNN	93.27	90.10	96.53
Huang et al. (2025)	2025	CNN-BiLSTM + STFFDA	92.42	92.42	NA
This work	2025	DWT + STD +PSD + MAV + Fuzzy entropy +CSAE-GRU	99.49	99.21	99.77

## 4 Conclusion

In this study, an advanced automatic detection method for epileptic seizure is introduced. To mitigate class imbalance commonly observed in EEG-based seizure datasets, we employ a novel two-stage data balancing strategy that combines cluster centroid-based under-sampling and BLSMOTE oversampling. Furthermore, we innovatively propose a hybrid classification model that combines CSAE with GRU network. By transferring the pre-trained sparse encoder weights to the classification stage, the model enhances the extraction of discriminative sparse features and improves generalization capability across diverse subjects. Extensive experiments conducted on both the Bonn and CHB-MIT datasets demonstrate that the proposed method achieves high accuracy, sensitivity, and specificity, outperforming conventional single-structure models. The framework is inherently simple to implement and operates independently of additional hyperparameter optimization strategies, thus reducing computational overhead and implementation complexity while maintaining superior detection performance.

## 5 Limitations and future work

Although the proposed CSAE-GRU framework has shown excellent performance in automatic seizure detection, several factors may limit its broader applicability. The experiments were conducted on two publicly available EEG datasets, which are widely used for benchmarking but do not fully capture the variability and artifacts typically present in clinical EEG recordings. In addition, the current work addresses only binary seizure detection, separating interictal and ictal conditions. In the next stage of our research, we aim to expand the model to classify multiple seizure types and to test it on larger, more heterogeneous clinical datasets. We also plan to design lighter network versions that can run efficiently in real time on portable or embedded EEG devices.

## Data Availability

Publicly available datasets were analyzed in this study. This data can be found here: The Bonn dataset is accessible at https://www.ukbonn.de/epileptologie/arbeitsgruppen/ag-lehnertz-neurophysik/downloads/, and the CHB-MIT dataset is available at https://physionet.org/content/chbmit/1.0.0/.

## References

[B1] Aayesha M. Bilal Qureshi M. Afzaal M. Qureshi S. Gwak J. (2022). Fuzzy-based automatic epileptic seizure detection framework. *Comput. Materials Continua* 70 5601–5630. 10.32604/cmc.2022.020348

[B2] Abdel Hady D. Mabrouk O. Abd El-Hafeez T. (2024). Employing machine learning for enhanced abdominal fat prediction in cavitation post-treatment. *Sci. Rep.* 14:11004. 10.1038/s41598-024-60387-x 38744923 PMC11094079

[B3] Acharya U. R. Hagiwara Y. Suren S. Isaev D. (2019). Maximyuk. Characterization of focal EEG signals: A review. *Future Generation Comput. Syst.* 91 290–299. 10.1016/j.future.2018.08.044

[B4] Al-Hadeethi H. Abdulla S. Diykh M. Green J. (2021). Determinant of covariance matrix model coupled with adaBoost classification algorithm for EEG seizure detection. *Diagnostics* 12:74. 10.3390/diagnostics12010074 35054242 PMC8774996

[B5] Al-Hadeethi H. Abdulla S. Diykh M. Deo R. C. Green J. H. (2020). Adaptive boost LS-SVM classification approach for time-series signal classification in epileptic seizure diagnosis applications. *Expert Syst. Appl.* 161:113676. 10.1016/j.eswa.2020.113676

[B6] Al-Hajjar A. Al-Qurabat A. (2023). An overview of machine learning methods in enabling IoMT-based epileptic seizure detection. *J. Supercomput.* 10.1007/s11227-023-05299-9 Online ahead of print. 37359338 PMC10123593

[B7] Ameen A. Fattoh I. E. Abd El-Hafeez T. Ahmed K. (2024). Advances in ECG and PCG-based cardiovascular disease classification: A review of deep learning and machine learning methods. *J. Big Data* 11:159. 10.1186/s40537-024-01011-7

[B8] Beniczky S. Wiebe S. Jeppesen J. Tatum W. Brazdil M. Wang Y. (2021). Automated seizure detection using wearable devices: A clinical practice guideline of the International league against epilepsy and the international federation of clinical neurophysiology. *Epilepsia* 62 632–646. 10.1111/epi.16818 33666944

[B9] Boonyakitanont P. Lek-uthai A. Chomtho K. Songsiri J. (2020). A review of feature extraction and performance evaluation in epileptic seizure detection using EEG. *Biomed. Signal Process. Control* 57:101702. 10.1016/j.bspc.2019.101702

[B10] Chen Z. Lu G. Xie Z. Shang W. (2020). A unified framework and method for EEG-based early epileptic seizure detection and epilepsy diagnosis. *IEEE Access.* 8 20080–20092. 10.1109/ACCESS.2020.2969055

[B11] Dissanayake T. Fernando T. Denman S. Sridharan S. Fookes C. (2021). Deep learning for patient-independent epileptic seizure prediction using scalp EEG signals. *IEEE Sensors J.* 21 9377–9388. 10.1109/JSEN.2021.3057076 34314363

[B12] Ein Shoka A. A. Dessouky M. M. El-Sayed A. El-Din Hemdan E. (2023). An efficient CNN based epileptic seizures detection framework using encrypted EEG signals for secure telemedicine applications. *Alexandria Eng. J.* 65 399–412. 10.1016/j.aej.2022.10.014

[B13] Eliwa E. Abd El-Hafeez T. (2025). Particle swarm optimization framework for Parkinson’s disease prediction. *PeerJ Comput. Sci.* 11:e3135. 10.7717/peerj-cs.3135 40989298 PMC12453757

[B14] Eliwa E. El Koshiry A. Abd El-Hafeez T. Farghaly H. M. (2023). Utilizing convolutional neural networks to classify monkeypox skin lesions. *Sci. Rep.* 13:14495. 10.1038/s41598-023-41545-z 37661211 PMC10475460

[B15] Eliwa E. Mohamed El Koshiry A. Abd El-Hafeez T. Omar A. (2024). Secure and transparent lung and colon cancer classification using blockchain and microsoft azure. *Adv. Respir. Med.* 92 395–420. 10.3390/arm92050037 39452059 PMC11505339

[B16] Fu R. Tian Y. Shi P. Bao T. (2020). Automatic detection of epileptic seizures in EEG using sparse CSP and fisher linear discrimination analysis algorithm. *J. Med .Syst.* 44:43. 10.1007/s10916-019-1504-1 31897615

[B17] Hamed B. A. Farghaly H. M. Omar A. Abd El-Hafeez T. (2025). Identifying key genetic variants in Alzheimer’s disease progression using Graph convolutional networks (GCN) and biological impact analysis. *J. Big Data* 12:171. 10.1186/s40537-025-01228-0

[B18] Hu D. Cao J. Lai X. Wang Y. Wang S. Ding Y. (2021). Epileptic state classification by fusing hand-crafted and deep learning EEG features. *IEEE Trans. Circuits Syst.* 68 1542–1546. 10.1109/TCSII.2020.3031399

[B19] Huang L. Zhou K. Chen S. Chen Y. Zhang J. (2024). Automatic detection of epilepsy from EEGs using a temporal convolutional network with a self-attention layer. *Biomed. Eng. Online* 23:50. 10.1186/s12938-024-01244-w 38824547 PMC11143608

[B20] Huang Z. Yang Y. Ma Y. Dong Q. Su J. Shi H. (2025). EEG detection and recognition model for epilepsy based on dual attention mechanism. *Sci. Rep.* 15:9404. 10.1038/s41598-025-90315-6 40108237 PMC11923361

[B21] Hussain W. Sadiq M. T. Siuly S. Rehman A. U. (2021). Epileptic seizure detection using 1 D-convolutional long short-term memory neural networks. *Appl. Acoustics* 177:107941. 10.1016/j.apacoust.2021.107941

[B22] Ingolfsson T. Benatti S. Wang X. Bernini A. Ducouret P. Ryvlin P. (2024). Minimizing artifact-induced false-alarms for seizure detection in wearable EEG devices with gradient-boosted tree classifiers. *Sci. Rep.* 14:2980. 10.1038/s41598-024-52551-0 38316856 PMC10844293

[B23] Islam M. Zhao X. Miao Y. Sugano H. Tanaka T. (2023). Epileptic seizure focus detection from interictal electroencephalogram: A survey. *Cogn. Neurodyn.* 17 1–23. 10.1007/s11571-022-09816-z 36704629 PMC9871145

[B24] Jindal K. Upadhyay R. Singh H. S. (2019). Application of tunable-Q wavelet transform based nonlinear features in epileptic seizure detection. *Analog Integrated Circuits Signal Process.* 100 437–452. 10.1007/s10470-019-01424-y

[B25] Khan K. A. Khan Y. U. Farooq O. (2020). A hybrid local binary pattern and wavelets based approach for EEG classification for diagnosing epilepsy. *Expert Syst. Appl.* 140:112895. 10.1016/j.eswa.2019.112895

[B26] Li M. Chen W. (2021). FFT-based deep feature learning method for EEG classification. *Biomed. Signal Process. Control* 66:102492. 10.1016/j.bspc.2021.102492

[B27] Lih O. Jahmunah V. Palmer E. Barua P. Dogan S. Tuncer T. (2023). EpilepsyNet: Novel automated detection of epilepsy using transformer model with EEG signals from 121 patient population. *Comput. Biol. Med.* 164:107312. 10.1016/j.compbiomed.2023.107312 37597408

[B28] Liu S. Wang J. Li S. Cai L. (2023). Epileptic seizure detection and prediction in EEGs using power spectra density parameterization. *IEEE Trans. Neural Syst. Rehabil. Eng.* 31 3884–3894. 10.1109/TNSRE.2023.3317093 37725738

[B29] Liu Y. Huang Y. Zhang X. Qi W. Guo J. Hu Y. (2020). Deep C-LSTM neural network for epileptic seizure and tumor detection using high-dimension EEG signals. *IEEE Access* 8 37495–37504. 10.1109/ACCESS.2020.2976156

[B30] Malekzadeh A. Zare A. Yaghoobi M. Kobravi H. Alizadehsani R. (2021). Epileptic seizures detection in EEG signals using fusion handcrafted and deep learning features. *Sensors* 21:7710. 10.3390/s21227710 34833780 PMC8624422

[B31] Mandhouj B. Cherni M. A. Sayadi M. (2021). An automated classification of EEG signals based on spectrogram and CNN for epilepsy diagnosis. *Analog. Integr. Circ. Sig. Process* 108 101–110. 10.1007/s10470-021-01805-2

[B32] Mekruksavanich S. Phaphan W. Jitpattanakul A. (2024). Epileptic seizure detection in EEG signals via an enhanced hybrid CNN with an integrated attention mechanism. *Math. Biosci. Eng.* 22 73–105. 10.3934/mbe.2025004 39949163

[B33] Mert A. Akan A. (2018). Seizure onset detection based on frequency domain metric of empirical mode decomposition. *SIViP* 12 1489–1496. 10.1007/s11760-018-1304-y

[B34] Mostafa G. Mahmoud H. Abd El-Hafeez T. ElAraby M. E. (2024). Feature reduction for hepatocellular carcinoma prediction using machine learning algorithms. *J. Big Data* 11:88. 10.1186/s40537-024-00944-3

[B35] Pale U. Teijeiro T. Rheims S. Ryvlin P. Atienza D. (2024). Combining general and personal models for epilepsy detection with hyperdimensional computing. *Artif. Intell. Med.* 148:102754. 10.1016/j.artmed.2023.102754 38325932

[B36] Peng H. Lei C. Zheng S. Zhao C. Wu C. Sun J. (2021). Automatic epileptic seizure detection via Stein kernel-based sparse representation. *Comput. Biol. Med.* 132:104338. 10.1016/j.compbiomed.2021.104338 33780870

[B37] Pidvalnyi I. Kostenko A. Sudakov O. Isaev D. (2025). Maximyuk. classification of epileptic seizures by simple machine learning techniques: Application to animals’ electroencephalography signals. *IEEE Access.* 13 8951–8962. 10.1109/ACCESS.2025.3527866

[B38] Raghu S. Sriraam N. Hegde A. S. Kubben P. L. (2019). A novel approach for classification of epileptic seizures using matrix determinant. *Expert Syst. Appl.* 127 323–341. 10.1016/j.eswa.2019.03.021

[B39] Ru Y. An G. Wei Z. Chen H. (2024). Epilepsy detection based on multi-head self-attention mechanism. *PLoS One* 19:e0305166. 10.1371/journal.pone.0305166 38861543 PMC11166279

[B40] Savadkoohi M. Oladunni T. Thompson L. A. (2020). machine learning approach to epileptic seizure prediction using Electroencephalogram (EEG) Signal. *Biocybern. Biomed. Eng.* 40 1328–1341. 10.1016/j.bbe.2020.07.004 36213693 PMC9540452

[B41] Sethy P. K. Panigrahi M. Vijayakumar K. Behera S. K. (2023). Machine learning based classification of EEG signal for detection of child epileptic seizure without snipping. *Int. J. Speech Technol.* 26 559–570. 10.1007/s10772-021-09855-7

[B42] Shah S. Y. Larijani H. Gibson R. M. Liarokapis D. (2024). Epileptic seizure classification based on random neural networks using discrete wavelet transform for electroencephalogram signal decomposition. *Appl. Sci.* 14:599. 10.3390/app14020599

[B43] Shankar A. Khaing H. K. Dandapat S. Barma S. (2021). Analysis of epileptic seizures based on EEG using recurrence plot images and deep learning. *Biomed. Signal Process. Control* 69:102854. 10.1016/j.bspc.2021.102854

[B44] Sharan R. Berkovsky S. (2020). Epileptic seizure detection using multi-channel EEG wavelet power spectra and 1-D convolutional neural networks. *Annu. Int. Conf. IEEE Eng. Med. Biol. Soc.* 2020 545–548. 10.1109/EMBC44109.2020.9176243 33018047

[B45] Shoeibi A. Ghassemi A. Alizadehsani R. Rouhani M. Khossravi A. Panahiazar M. (2021). A comprehensive comparison of handcrafted features and convolutional autoencoders for epileptic seizures detection in EEG signals. *Expert Syst. Appl.* 163:113788. 10.1016/j.eswa.2020.113788

[B46] Srinivasan S. Dayalane S. Mathivanan S. Rajadurai H. Jayagopal P. Dalu G. (2023). Detection and classification of adult epilepsy using hybrid deep learning approach. *Sci. Rep.* 13:17574. 10.1038/s41598-023-44763-7 37845403 PMC10579259

[B47] Supriya S. Siuly S. Wang H. Zhang Y. (2020). Automated epilepsy detection techniques from electroencephalogram signals: A review study. *Health Inf. Sci. Syst.* 8:33. 10.1007/s13755-020-00129-1 33088489 PMC7550618

[B48] Takahashi H. Emami A. Shinozaki T. Kunii N. Matsuo T. Kawai K. (2020). Convolutional neural network with autoencoder-assisted multiclass labelling for seizure detection based on scalp electroencephalography. *Comput. Biol. Med.* 125:104016. 10.1016/j.compbiomed.2020.104016 33022521

[B49] Tang Y. Wu Q. Mao H. Guo L. (2024). Epileptic seizure detection based on path signature and Bi-LSTM network with attention mechanism. *IEEE Trans. Neural Syst. Rehabil. Eng.* 32 304–313. 10.1109/TNSRE.2024.3350074 38224524

[B50] Tian X. Deng Z. Ying W. Choi K. Wu D. Qin B. (2019). Deep multi-view feature learning for EEG-based epileptic seizure detection. *IEEE Trans. Neural Syst. Rehabil. Eng.* 27 1962–1972. 10.1109/TNSRE.2019.2940485 31514144

[B51] Tran L. Tran H. Le T. Huynh T. Tran H. Dao S. (2022). Application of machine learning in epileptic seizure detection. *Diagnostics* 12:2879. 10.3390/diagnostics12112879 36428941 PMC9689720

[B52] Tsipouras M. G. (2019). Spectral information of EEG signals with respect to epilepsy classification. *EURASIP J. Adv. Signal Process.* 1:10. 10.1186/s13634-019-0606-8

[B53] van Westrhenen A. Lazeron R. van Dijk J. Leijten F. Thijs R. (2023). Multimodal nocturnal seizure detection in children with epilepsy: A prospective, multicenter, long-term, in-home trial. *Epilepsia* 64 2137–2152. 10.1111/epi.17654 37195144

[B54] Wang X. Wang Y. Liu D. Wang Y. Wang Z. (2023). Automated recognition of epilepsy from EEG signals using a combining space-time algorithm of CNN-LSTM. *Sci. Rep.* 13:14876. 10.1038/s41598-023-41537-z 37684278 PMC10491650

[B55] Wang Z. Li S. Wu D. (2025). Canine EEG helps human: Cross-species and cross-modality epileptic seizure detection via multi-space alignment. *Natl. Sci. Rev.* 12:nwaf086. 10.1093/nsr/nwaf086 40330047 PMC12051906

[B56] Wei B. Zhao X. Shi L. Xu L. Liu T. Zhang J. (2021). A deep learning framework with multi-perspective fusion for interictal epileptiform discharges detection in scalp electroencephalogram. *Neural Eng.* 18:ac0d60. 10.1088/1741-2552/ac0d60 34157696

[B57] Wei Z. Zou J. Zhang J. Xu J. (2019). Automatic epileptic EEG detection using convolutional neural network with improvements in time-domain. *Biomed. Signal Process. Control* 53:101551. 10.1016/j.bspc.2019.04.028

[B58] Yang X. Zhao J. Sun Q. Lu J. Ma X. (2021). An effective dual self-attention residual network for seizure prediction. *IEEE Trans. Neural Syst. Rehabil. Eng.* 29 1604–1613. 10.1109/TNSRE.2021.3103210 34370668

[B59] Yogarajan G. Alsubaie N. Rajasekaran G. Revathi T. Alqahtani M. Abbas M. (2023). EEG-based epileptic seizure detection using binary dragonfly algorithm and deep neural network. *Sci. Rep.* 13:17710. 10.1038/s41598-023-44318-w 37853025 PMC10584945

[B60] Yu H. Meng X. (2023). Characteristic analysis of epileptic brain network based on attention mechanism. *Sci. Rep.* 13:10742. 10.1038/s41598-023-38012-0 37400535 PMC10317957

[B61] Zanetti R. Aminifar A. Atienza D. (2020). Robust epileptic seizure detection on wearable systems with reduced false-alarm rate. *Annu. Int. Conf. IEEE Eng. Med. Biol. Soc.* 2020 4248–4251. 10.1109/EMBC44109.2020.9175339 33018934

[B62] Zhang L. Wang X. Jiang J. Xiao N. Guo J. Zhuang K. (2023). Automatic interictal epileptiform discharge (IED) detection based on convolutional neural network (CNN). *Front. Mol. Biosci.* 10:1146606. 10.3389/fmolb.2023.1146606 37091867 PMC10119410

[B63] Zhang Y. Yao S. Yang R. Liu X. Qiu W. Han L. (2022). Epileptic seizure detection based on bidirectional gated recurrent unit network. *IEEE Trans. Neural Syst. Rehabil. Eng.* 30 135–145. 10.1109/TNSRE.2022.3143540 35030083

[B64] Zhang Z. Xiao M. Ji T. Jiang Y. Lin T. Zhou X. (2024). Efficient and generalizable cross-patient epileptic seizure detection through a spiking neural network. *Front. Neurosci.* 17:1303564. 10.3389/fnins.2023.1303564 38268711 PMC10805904

